# X-ray based radiomics machine learning models for predicting collapse of early-stage osteonecrosis of femoral head

**DOI:** 10.1038/s41598-025-94878-2

**Published:** 2025-04-20

**Authors:** Yaqing He, Yang Chen, Yusen Chen, Pingshi Li, Le Yuan, Maoxiao Ma, Yuhao Liu, Wei He, Wu Zhou, Leilei Chen

**Affiliations:** 1 The Third Clinical Medical College of Guangzhou University of Chinese Medicine, Guangzhou, Guangdong People’s Republic of China; 2 School of Medical Information Engineering, Guangzhou University of Chinese Medicine, 232 Wide Ring East Road, Panyu District, Guangzhou, 510006 Guangdong People’s Republic of China; 3 Department of Orthopaedics, The First Affiliated Hospital of Guangzhou University of Chinese Medicine, Guangzhou, Guangdong People’s Republic of China; 4 Traumatology and Orthopaedics Institute, Guangzhou University of Chinese Medicine, 261 Longxi Avenue, Liwan District, Guangzhou, 510378 Guangdong People’s Republic of China; 5 Department of Orthopaedics, The Third Affiliated Hospital of Guangzhou University of Chinese Medicine, Guangzhou, Guangdong People’s Republic of China

**Keywords:** Radiomics, Machine learning, Artificial intelligence, Osteonecrosis of the femoral head, X-ray, Diseases, Medical research

## Abstract

This study aimed to develop an X-ray radiomics model for predicting collapse of early-stage osteonecrosis of the femoral head (ONFH). A total of 87 patients (111 hips; training set: *n* = 67, test set: *n* = 44) with non-traumatic ONFH at Association Research Circulation Osseous (ARCO) stage II were retrospectively enrolled. Following data dimensionality reduction and feature selection, radiomics models were constructed based on anteroposterior (AP), frog-lateral (FL), and AP + FL combined view using random forest (RF), support vector machine (SVM), and stochastic gradient descent (SGD). After the optimal radiomics model was selected based on areas under the curve (AUC), its performance on the test set was compared with that of orthopaedists using receiver operating characteristic (ROC) curves and confusion matrices. Among all radiomics models, the SVM-based AP + FL combined view model (AP + FL-Rad_SVM) achieved the highest individual performance demonstrating an AUC of 0.904 (95% CI 0.829 –0.978) in the test set, which was significantly better than that of three attending surgeons (*p* = 0.014, 0.004, and 0.045, respectively). The SVM model based on AP + FL views of hip X-ray exhibited excellent ability in predicting the collapse of ONFH and showed superior performance compared with less experienced orthopaedic surgeons. This model may inform clinical decision-making for early-stage ONFH.

**Supplementary Information:**

The online version contains supplementary material available at 10.1038/s41598-025-94878-2.

## Introduction

Osteonecrosis of the femoral head (ONFH) is a progressive and disabling orthopaedic disease that affects patients of all ages, particularly young adults^1^. Femoral head collapse represents a critical prognostic determinant in the clinical progression of ONFH. Cumulative evidence suggests^2–5^ that nearly half of ONFH patients will develop femoral head collapse within 2 years. For femoral heads unlikely to collapse in the short term, more conservative treatments are preferred; for those at risk of collapse, various hip-preserving treatments can achieve long-term satisfactory outcomes before collapse occurs. Once collapse occurs, most hips progress to joint destruction or osteoarthritis, ultimately requiring joint replacement and increasing the risk of future revision hip arthroplasty^6^. In this context, predicting individual outcomes is crucial for patients with early ONFH. However, no widely recognized prognostic system can accurately and efficiently predict femoral head collapse.

Imaging examination is a vital method for diagnosing and analyzing ONFH. X-ray anteroposterior (AP) and frog-leg lateral (FL) views can provide comprehensive information about the anterior and lateral bone structures, which are critical weight-bearing areas of the femoral head and contain important prognostic information, such as the location, size, morphology, and boundary of the necrotic lesion^7–10^. However, quantitative assessment of these parameters lacks standardization, and none can accurately predict collapse alone. Furthermore, interobserver variability among clinicians, it is challenging to achieve an objective and unified evaluation of femoral head collapse outcomes.

With the rapid advancement of technology, radiomics—an emerging artificial intelligence technique—can provide comprehensive and objective imaging information through the high-throughput extraction and mining of large numbers of images. Several studies^11–14^ have proposed machine learning systems based on radiomics models to automate the diagnosis and staging of ONFH. However, due to the uncertainty of collapse prediction, a substantial proportion of patients receive unnecessarily aggressive interventions in the early disease stage, resulting in a limited sample size of non-surgical patients completing long-term follow-up and few researchers focusing on predicting collapse. Given the crucial role of femoral head collapse in selecting early ONFH treatments, we developed several machine learning models based on radiomics analysis of X-ray images to achieve objective, efficient, and accurate prediction of collapse in early ONFH and assist surgeons in planning clinical treatment.

## Materials and methods

### Ethics statement

This study adhered to the Declaration of Helsinki, registered with the Chinese Clinical Trial Registry (chictr.org.cn; Registration ID: ChiCTR2400085757), and approved by the Institutional Review Board (Approval No.: PJ-XS-20240513-003). Given the retrospective nature of the study and the use of anonymized data, the requirement for patient approval or written informed consent for reviewing medical records or images was waived.

### Patients

This retrospective study included patients with Association Research Circulation Osseous (ARCO) stage II ONFH recruited from two tertiary referral centers from January 2019 to December 2023. Inclusion criteria comprised: (1) ARCO stage II ONFH confirmed by MRI imaging in these two hospitals or other medical institutions; (2) patients aged 18–65 years; (3) hips without surgical interventions within the 2-year follow-up or before femoral head collapse. The exclusion criteria were as follows: (1) patients with other hip diseases, such as developmental dysplasia of the hip, femoroacetabular impingement syndrome, tumors, or diseases affecting bone metabolism; (2) previous history of hip trauma or hip surgery; (3) incomplete follow-up imaging data or X-ray radiographs that did not meet the stringent and standard AP and FL views. The diagnosis of ONFH was based on the updated Association Research Circulation Osseous (ARCO) grading system^15^.

**Fig. 1:**
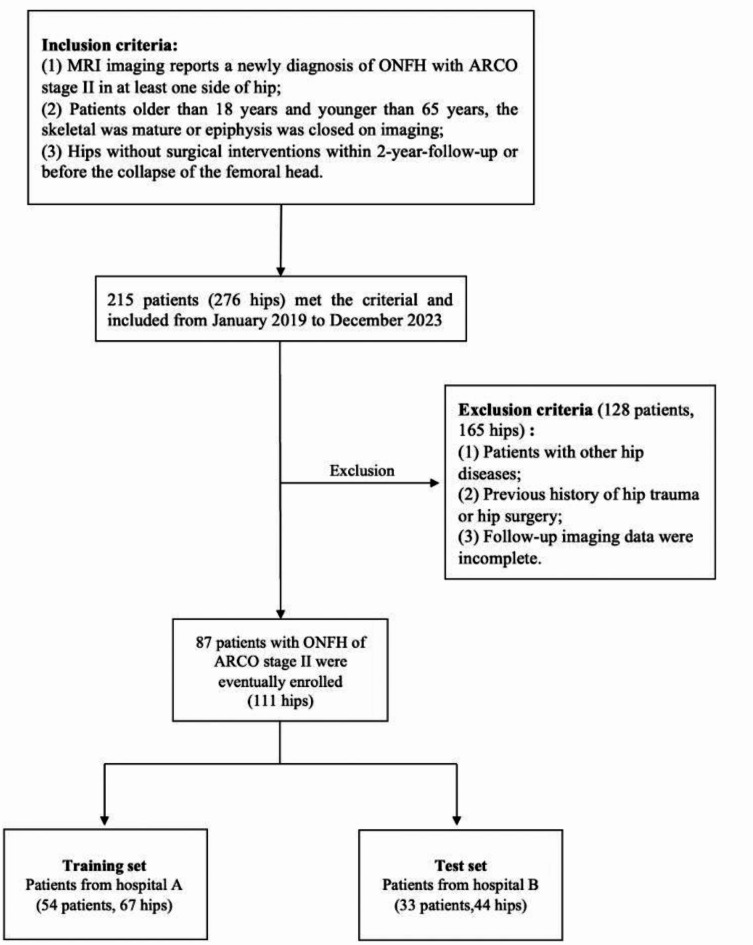
Flowchart for selection of case with early stage ONFH. ONFH: osteonecrosis of femoral head. ARCO: Association Research Circulation Osseous.

This study used the hip as the unit of analysis. Ultimately, a total of 87 patients with 111 hips in ARCO stage II at initial diagnosis were included in this retrospective cohort study. The case selection process is shown in Fig. 1. After including cases that met the criteria, these hips were divided into training and test sets based on the hospitals from which the cases were collected. A total of 67 hips from hospital A were included in the training set, and 44 hips from hospital B were included in the test set.

### Image acquisition and annotation

All images were obtained in standard AP and FL views. Standard AP views were taken with the patient in a supine position, centered on the midpoint between the anterior superior iliac spine and the pubic symphysis, with both legs internally rotated by 15°. For standard FL views, patients in a supine position with both hips flexed at 30°, thighs abducted and externally rotated, and knees flexed, with the plantar surfaces opposed. The subjects’ archived images were exported and saved in the Digital Imaging and Communications in Medicine (DICOM) format.

To ensure reliability, all images were anonymized in batches by removing basic information, such as patient names and identification numbers. Two deputy chief orthopaedic surgeons with 20 years of experience independently reviewed the included X-ray images and manually outlined the region of interest (ROI) representing necrotic tissue in the femoral head in each AP and FL view using the open-source ITK-SNAP software (version 4.0.1, http://www.itksnap.org/). To ensure the reliability of the results, any discrepancies in the delineated ROIs between the two surgeons were resolved through discussion and negotiation, with reference to the corresponding MRI sequences. The outcome was defined as visible subchondral fracture or collapse in the AP or FL views of X-ray during the 2-year follow-up, or performance of total hip arthroplasty.

### Image preprocessing

To ensure the precision and reliability of radiomics feature extraction, a standardized preprocessing protocol for medical images was implemented. Histogram discretization was employed to mitigate the influence of image noise on feature extraction and reduce computational complexity. Subsequently, Gaussian kernels with standard deviations (σ) of 1.0, 2.0, and 3.0 were used for multi-scale smoothing to further diminish image noise. To standardize voxel spacing across all images, resampling was performed to adjust the voxel dimensions to 1 mm × 1 mm.

### Radiomics features extraction and radiomics model construction

All processes were conducted in Python (version 3.7; https://www.python.org) with the following key libraries: Pyradiomics (version 3.1.0; http://pypi.org/project/Pyradiomics) for radiomics feature extraction, NumPy (version 1.23.4) for numerical operations, Pandas (version 1.5.3) for data processing, Scikit-learn (version 1.5.2) for machine learning model development, Matplotlib (version 3.3.4) and Seaborn (version 0.11.1) for data visualization, and SHAP (version 0.43.0) for interpreting model predictions. The hardware configuration included a motherboard model 8DMF143, an Intel (Xeon) Gold 5218 processor (CPU) @ 2.30 GHz, dual NVIDIA RTX 2080 Ti GPUs (11GB VRAM each), and 64.0GB of RAM (model 36ASF4G72PZ-2G6D1).

Radiomics features (Supplementary Tab. S1 online), including shape-based features, intensity features, and texture features (divided into gray level co-occurrence matrix [GLCM], gray level size zone matrix [GLSZM], gray level run length matrix [GLRLM], neighboring gray tone difference matrix [NGTDM], and gray level dependence matrix [GLDM] features) were extracted from the preprocessed images of the AP and FL views, respectively. Considering that combining the AP and FL views provides a more comprehensive observation for the femoral head’s three-dimensional spherical structure, especially changes in the anterior and lateral parts, we also combined the features of both views.

The least absolute shrinkage and selection operator (LASSO) regression algorithm was used to screen important features from the extracted feature sets. By constructing a penalty function (λ), some regression coefficients were shrunk to force unimportant features to become 0, while stable features were incorporated into LASSO analysis. Based on the minimum standard, 10-fold cross-validation was used to determine the optimal λ value, and the corresponding model was used to screen non-zero coefficient features to obtain independent and stable features.

Feature normalization was achieved through Z-score standardization before feature modeling. To select a classifier model with optimal prognostic performance for collapse, our study selected three mainstream machine learning algorithms—random forest (RF), support vector machine (SVM), and stochastic gradient descent (SGD)—to construct the radiomics models. In order to avoid overfitting, Hyperparameter tuning employed Bayesian optimization with 5-fold cross-validation was used in the training set to select the optimal parameters of these classification models (Supplementary Tab. S2 online). The performances of different classification models were compared.

Model performance was quantified using accuracy, precision, recall(sensitivity), specificity, and F1-score on both training and test sets. These matrices are defined as follows:

1 $$Accuracy=\frac{TP+TN}{TP+TN+FP+FN}$$

2 $$Precision=\frac{TP}{TP+FP}$$

3 $$Recall=\frac{TP}{TP+FN}$$

4 $$\text{S}\text{p}\text{e}\text{c}\text{i}\text{f}\text{i}\text{c}\text{i}\text{t}\text{y}=\frac{TN}{TN+FP}$$

5 $$F1=2{*}\frac{Precision{*}Recall}{Precision+Recall}$$

TP, FP, TN, and FN represent true positives, false positives, true negatives, and false negatives, respectively. Accuracy is defined as the proportion of correctly classified collapsed and non-collapsed cases among all samples. Precision measures the proportion of correctly classified collapsed cases among all predicted collapsed cases. Recall, also known as sensitivity, indicates the proportion of correctly predicted collapsed cases among all actual collapsed samples. Specificity is the proportion of correctly predicted non-collapsed cases among all actual non-collapsed samples. The F1-score is a balanced metric for assessing the classification model’s performance, considering both precision and recall, and can be considered the harmonic average of these two measures.

**Fig. 2:**
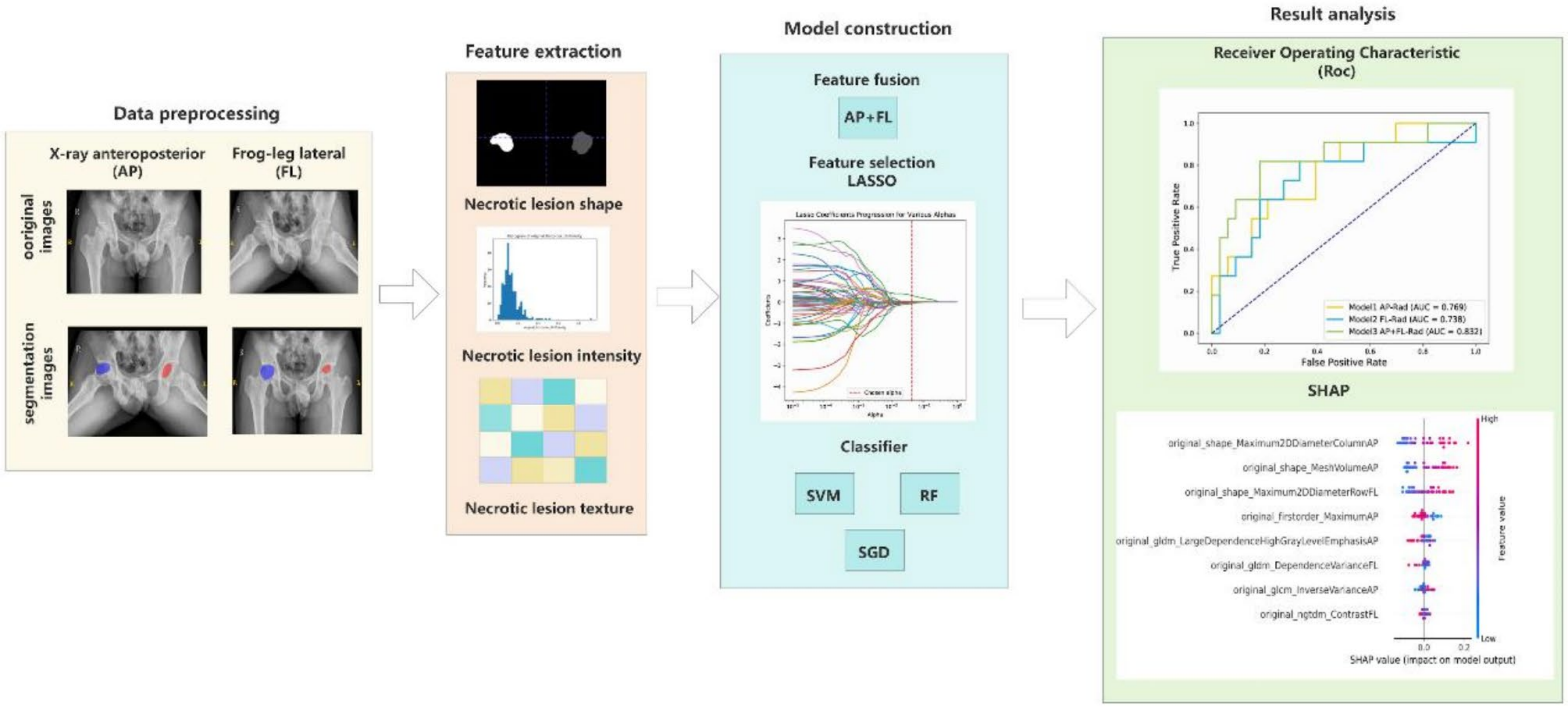
Flowchart for building and validating predictive models. Schematic overview of the prediction of collapse with our model. Radiographs from AP and FL views were obtained, and ROIs were delineated; then the radiomics features were extracted from the preprocessed images; after the LASSO regression algorithm was used to screen important features, SVM, RF, and SGD were used to construct the radiomics models; the discriminative power of these models was compared using ROC curves, and SHAP values were applied to explain the importance of each feature. AP: anteroposterior. FL: frog-leg lateral. ROI: region of interest. LASSO: least absolute shrinkage and selection operator. SVM: support vector machine. RF: random forest. SGD: stochastic gradient descent. ROC: receiver operating characteristic.

The discriminative power of these three machine-learning models was compared using receiver operating characteristic (ROC) curves and the area under the ROC curve (AUC). Calibration curves were applied to evaluate the clinical value of the models. And interpretable SHAP plots were used to interpret the relationship between predictors and outcomes in the optimal machine-learning model. The overall study flowchart is shown in Fig. 2.

### Comparison with manual identification

To compare the predictive ability of the machine learning model with manual identification, 44 hips from the test subset were reviewed by our manual identification group, which consisted of six orthopaedic surgeons blinded to data collection and analysis (three resident surgeons with 5 years of experience in bone and joint disorders and three attending surgeons with 10 years of experience). Using standardized criteria from established prognostic systems^4,16–19^ (e.g., radiographic stage, lesion size, necrosis location, anterior and lateral preserved angle), the surgeons were asked to independently predict the collapse of each femoral head within 2 years based solely on plain radiographs. The prediction results derived from the optimal model and the orthopaedic surgeons were compared.

The accuracy, sensitivity, and specificity of evaluations by each orthopaedic surgeon were calculated, as well as the consistency among surgeons. Additionally, confusion matrices, ROC curves, and AUC were applied to compare the predicted results from the orthopaedic surgeons and the radiomics model.

### Statistical analysis

All statistical analyses were performed using SPSS (version 26.0.0 IBM), Python (version 3.7; https://www.python.org), and R statistical software (version 4.4.0; https://www.r-project.org).

The calculation of model performance metrics, including AUC, accuracy, precision, recall, and F1-score, was implemented using Python. For comparison of baseline data and the evaluation of the machine learning model versus surgeon predictions, relevant statistical packages in R were used. Continuous variables in the baseline data were reported as medians and interquartile ranges (IQR), with comparisons between groups performed using the Mann-Whitney U test. Categorical data were evaluated using the chi-square test. DeLong’s test was used to determine the significance of differences in AUC between machine-learning model and the orthopaedic surgeons. Statistical significance was defined as α = 0.05 (two-sided). Cohen’s kappa analysis was conducted using SPSS to assess the agreement among surgeons regarding the prediction of femoral head collapse.

## Results

### Patients’ characteristics

The study enrolled 111 hips (87 patients; 31 females and 56 males). The affected hips were divided into training and test subsets based on their sources, and the demographic features of these two groups are described in Table 1. No significant inter-group differences were observed in baseline characteristics (all *p* > 0.05).

**Table 1:** Demographic and clinical characteristics of included patients.

Interrelating parameter		Overall (*n* = 87 patients/111 hips)	Training subset (*n* = 54 patients/67 hips)	Test subset (*n* = 33 patients/44 hips)	U/χ^2^	*p*-value
Age (year)		36.0 [30.0, 45.0]	35.0 [30.0, 50.0]	36.5 [31.0, 43.0]	*U* = 1527.50	0.75^†^
Gender	Men	56 (64.4%)	37 (68.5%)	19 (57.6%)	χ^2^ = 1.07	0.30^‡^
	Women	31 (35.6%)	17 (31.5%)	14 (42.4%)		
Affected hip side	Left	57 (51.4%)	34 (50.7%)	23 (52.3%)	χ^2^ = 0.03	0.88^‡^
	Right	54 (48.6%)	33 (49.3%)	21 (47.7%)		
Associated factor	Corticosteroid	58 (52.3%)	33 (49.3%)	25 (56.8%)	χ^2^ = 3.62	0.16^‡^
	Alcohol	40 (36.0%)	23 (34.3%)	17 (38.6%)		
	Idiopathic	13 (11.7%)	11 (16.4%)	2 (4.55%)		
Outcome	Collapsed	37 (33.3%)	26 (38.8%)	11 (25.0%)	χ^2^ = 2.28	0.13^‡^
	Non-collapsed	74 (66.7%)	41 (61.2%)	33 (75.0%)		

^†^: Non-parametric Mann-Whitney U test rank sum test;

^‡^: Pearson ‘s chi-squared test.

### Prediction performance of the radiomics signature models

**Fig. 3:**
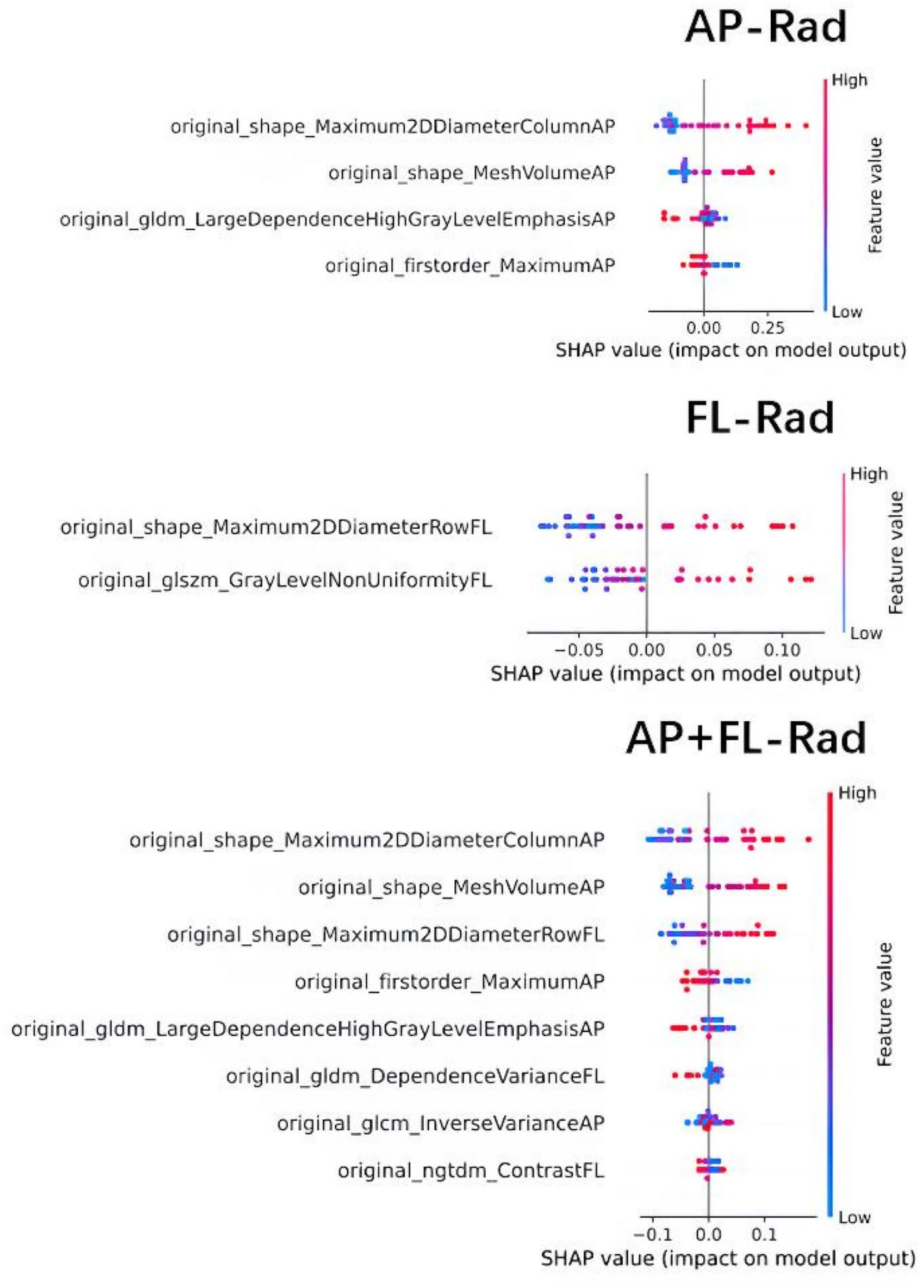
The best image radiomics features and corresponding SHAP values were selected based on the multi-view AP and FL radiomics features and their combined (AP + FL) features. Each row on the vertical axis represents a radiomics feature, while the horizontal axis indicates the SHAP value. Each dot represents a sample. Points were generated based on the SHAP values of each feature for the affected hips. The color of the points represents the contribution of radiomics features to the prediction results of the classifier model. Red indicates a higher eigenvalue, while blue indicates a lower eigenvalue. The probability of femoral head collapse increased with the increase in the feature’s SHAP value. AP-Rad: AP view model radiomics feature datasets. FL-Rad: FL view model radiomics feature datasets. AP + FL-Rad: AP + FL combined view model radiomics feature datasets.

Radiomics features were extracted from AP and FL view X-ray images, with 105 initial features retained for each view. Radiomics feature datasets were then constructed for the AP, FL, and AP + FL combined models (AP-Rad, FL-Rad, and AP + FL-Rad). Among these, LASSO regularization retained 4 (AP), 2 (FL), and 8 (AP + FL) discriminative features, respectively (Fig. 3). The SHAP summary dot plot depicted the global model interpretation using the SHAP method and ranked the features by their importance in predicting the collapse. According to the results, the features with the highest SHAP values in AP view are original_shape_Maximum2DDiameterColumn, original_shape_MeshVolume, and original_gldm_LargeDependenceHighGrayLevelEmphasis. The most influential features in the FL view are original_shape_Maximum2DDiameterRow and original_glszm_GrayLevelNonUniformity. The most impactful features in the AP + FL combined view include original_shape_Maximum2DDiameterColumn (AP view), original_shape_MeshVolume (AP view), original_shape_Maximum2DDiameterRow (FL view), and original_firstorder_Maximum (AP view).

**Fig. 4:**
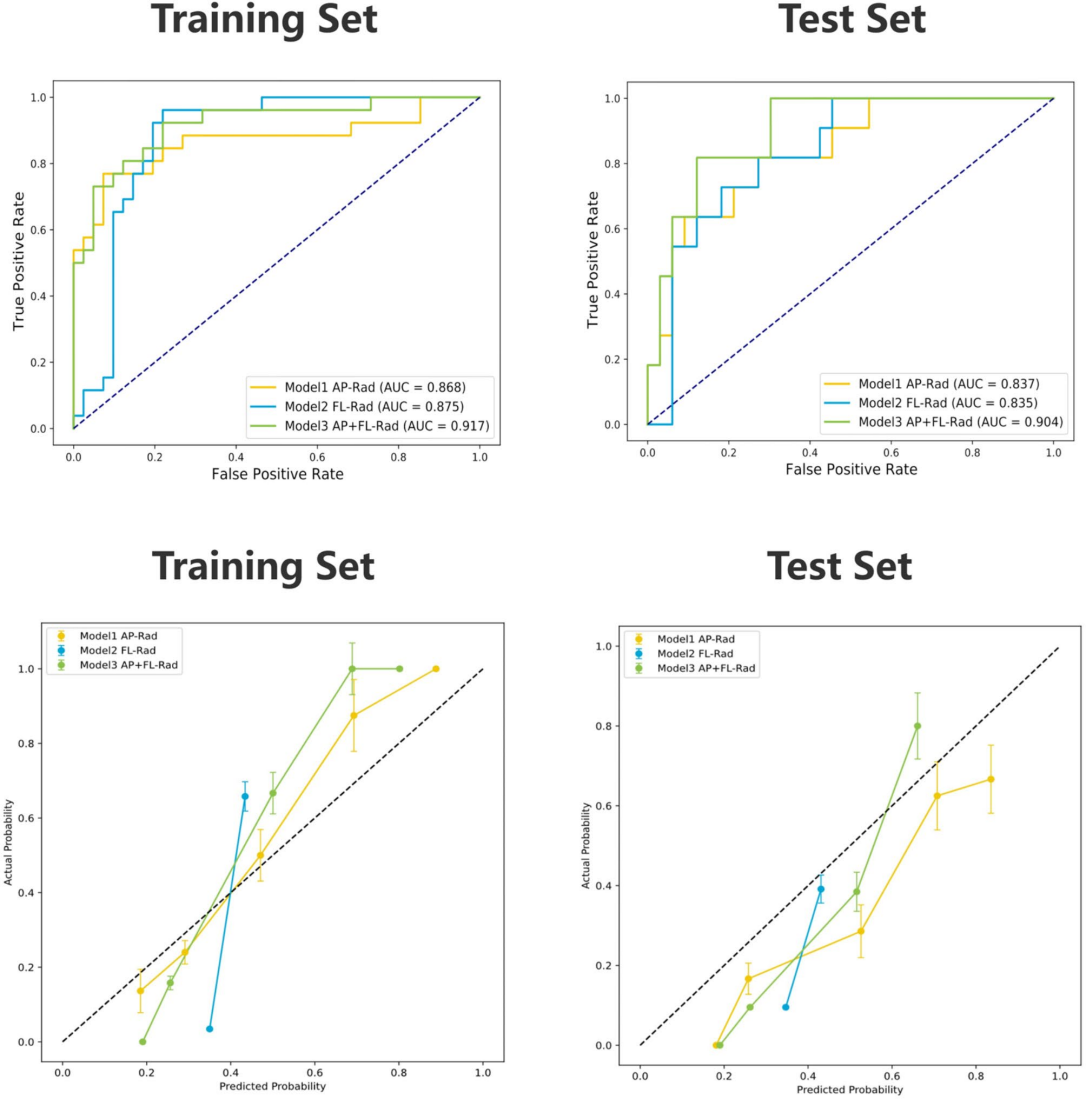
Performance of AP-Rad model, FL-Rad model and AP + FL-Rad model under SVM in predicting the collapse of ONFH. Model performance in the training and test sets is demonstrated using Receiver Operating Characteristics (ROC) curves. The vertical axis represents the True Positive Rate (TPR), while the horizontal axis represents the False Positive rate (FPR). The FPR and TPR values corresponding to each threshold were plotted as coordinate points and connected to form the ROC curve. Calibration curves of SVM models under different modes in the training and test sets. The y-axis represents the actual probability of collapse, while the x-axis represents the predicted probability of collapse. A dashed line with a slope of 1 represents the ideal calibration curve, indicating perfect agreement between predicted and actual probabilities. The closer the model’s predicted calibration curve is to the ideal calibration curve, the better the model’s prediction performance. AP-Rad: AP view model radiomics feature datasets. FL-Rad: FL view model radiomics feature datasets. AP + FL-Rad: AP + FL combined view model radiomics feature datasets.

In the training dataset, all radiomics models performed well. However, in the test set, the SVM classifier in the AP + FL-Rad mode achieved the best comprehensive prediction performance with an AUC of 0.904, higher than that of RF (0.826) and SGD (0.813) in the same mode (Table 2). Additionally, the AP + FL combined mode consistently showed better comprehensive performance than the single AP or FL modes across these classifier models. For the SVM model, the AUC of the AP + FL combined mode was higher than that of the single AP or FL model (0.917 vs. 0.868 vs. 0.875 in the training set and 0.904 vs. 0.837 vs. 0.835 in the test set). Figure 4a shows the ROC curves and AUC analysis for the training and test sets in AP, FL, and AP + FL modes under the SVM model.

**Table 2:** The effectiveness of each model in predicting the collapse of ONFH.

Sequence	Classifier	AUC(95%CI)	Accuracy	Precision	Recall/sensitivity	Specificity	F1-score
Training set							
AP-Rad	SVM	0.868(0.799 ~ 0.936)	0.821	0.938	0.577	0.976	0.714
FL-Rad	SVM	0.875(0.842 ~ 0.909)	0.806	0.741	0.769	0.829	0.755
AP + FL-Rad	SVM	0.917(0.852 ~ 0.983)	0.866	0.905	0.731	0.951	0.809
AP-Rad	RF	0.896(0.839 ~ 0.953)	0.836	0.857	0.692	0.927	0.766
FL-Rad	RF	0.932(0.862 ~ 1.000)	0.851	0.808	0.808	0.878	0.808
AP + FL-Rad	RF	0.952(0.884 ~ 1.000)	0.851	0.864	0.731	0.927	0.792
AP-Rad	SGD	0.872(0.821 ~ 0.924)	0.791	0.800	0.615	0.897	0.696
FL-Rad	SGD	0.796(0.740 ~ 0.853)	0.701	0.625	0.577	0.780	0.600
AP + FL-Rad	SGD	0.886(0.828 ~ 0.943)	0.836	0.857	0.692	0.927	0.766
Test set							
AP-Rad	SVM	0.837(0.753 ~ 0.922)	0.841	0.700	0.636	0.909	0.667
FL-Rad	SVM	0.835(0.791 ~ 0.878)	0.773	0.538	0.636	0.818	0.583
AP + FL-Rad	SVM	0.904(0.829 ~ 0.978)	0.795	0.563	0.818	0.788	0.667
AP-Rad	RF	0.796(0.721 ~ 0.871)	0.750	0.500	0.636	0.788	0.560
FL-Rad	RF	0.784(0.708 ~ 0.859)	0.773	0.538	0.636	0.818	0.583
AP + FL-Rad	RF	0.826(0.746 ~ 0.907)	0.727	0.471	0.727	0.727	0.571
AP-Rad	SGD	0.802(0.734 ~ 0.869)	0.773	0.533	0.727	0.788	0.615
FL-Rad	SGD	0.747(0.676 ~ 0.817)	0.795	0.583	0.636	0.848	0.609
AP + FL-Rad	SGD	0.813(0.740 ~ 0.885)	0.818	0.600	0.818	0.818	0.692

AP-Rad: AP view model radiomics feature datasets.

FL-Rad: FL view model radiomics feature datasets.

AP + FL-Rad: AP + FL combined view model radiomics feature datasets.

SVM: support vector machine.

RF: random forest.

SGD: stochastic gradient descent.

AUC: areas under the receiver operating characteristic curve.

CI: confidence interval.

The AP + FL-Rad SVM model demonstrated superior discriminative ability (AUC = 0.904, 95%CI 0.829–0.978) with 81.8% sensitivity on the external test set. Although its calibration performance on the training set was slightly lower than that of the AP-Rad model, its probability predictions were better aligned with observed outcomes in the test set (Fig. 4b), indicating robustness to data distribution shifts across varied scenarios.

To ensure stability and sustainability of radiomics model, we ultimately selected the SVM model in AP + FL combined mode as the optimal radiomics model. We also explored the marginal effects of the optimal radiomics model’s features and the correlation between collapse risk and radiomics features (Supplementary Fig. S1 online).

### Comparison between machine-learning model and manual recognition

To compare the predictive performance of machine-learning models and manual recognition, we used test set to assess their ability to predict collapse. The individual results examined by six orthopaedists are shown in Table 3. The consistency of predicted results among orthopaedists showed substantial variation, and there was a large difference between their predicted results and the actual outcome (Table 4).

**Table 3:** The performance of 6 orthopaedists on collapse prediction.

	AUC(95%CI)	Accuracy	Sensitivity	Specificity
Surgeon 1	0.636(0.464 ~ 0.809)	0.682	0.546	0.727
Surgeon 2	0.606(0.434 ~ 0.778)	0.591	0.636	0.576
Surgeon 3	0.652(0.489 ~ 0.814)	0.614	0.727	0.576
Surgeon 4	0.500(0.342 ~ 0.658)	0.614	0.273	0.727
Surgeon 5	0.500 (0.323 ~ 0.677)	0.477	0.546	0.455
Surgeon 6	0.545(0.384 ~ 0.707)	0.455	0.727	0.364

AUC: areas under the receiver operating characteristic curve.

CI: confidence interval.

**Table 4:** Interrater reliability presented with Cohen’s kappa analysis among the orthopaedic surgeons and actual outcome.

		Attending surgeons			Resident surgeons			Actual outcome
		1	2	3	4	5	6	
Attending surgeons	1		0.447	0.318	0.097	0.249	− 0.073	0.243
	2	0.447		0.318	− 0.160	0.140	− 0.165	0.163
	3	0.318	0.318		0.091	0.000	− 0.136	0.227
Resident surgeons	4	0.097	-0.160	0.091		0.127	0.007	0.000
	5	0.249	0.140	0.000	0.127		0.017	0.000
	6	− 0.073	-0.165	-0.136	0.007	0.017		0.059
Actual outcome		0.243	0.163	0.227	0.000	0.000	0.059	

**Fig. 5:**
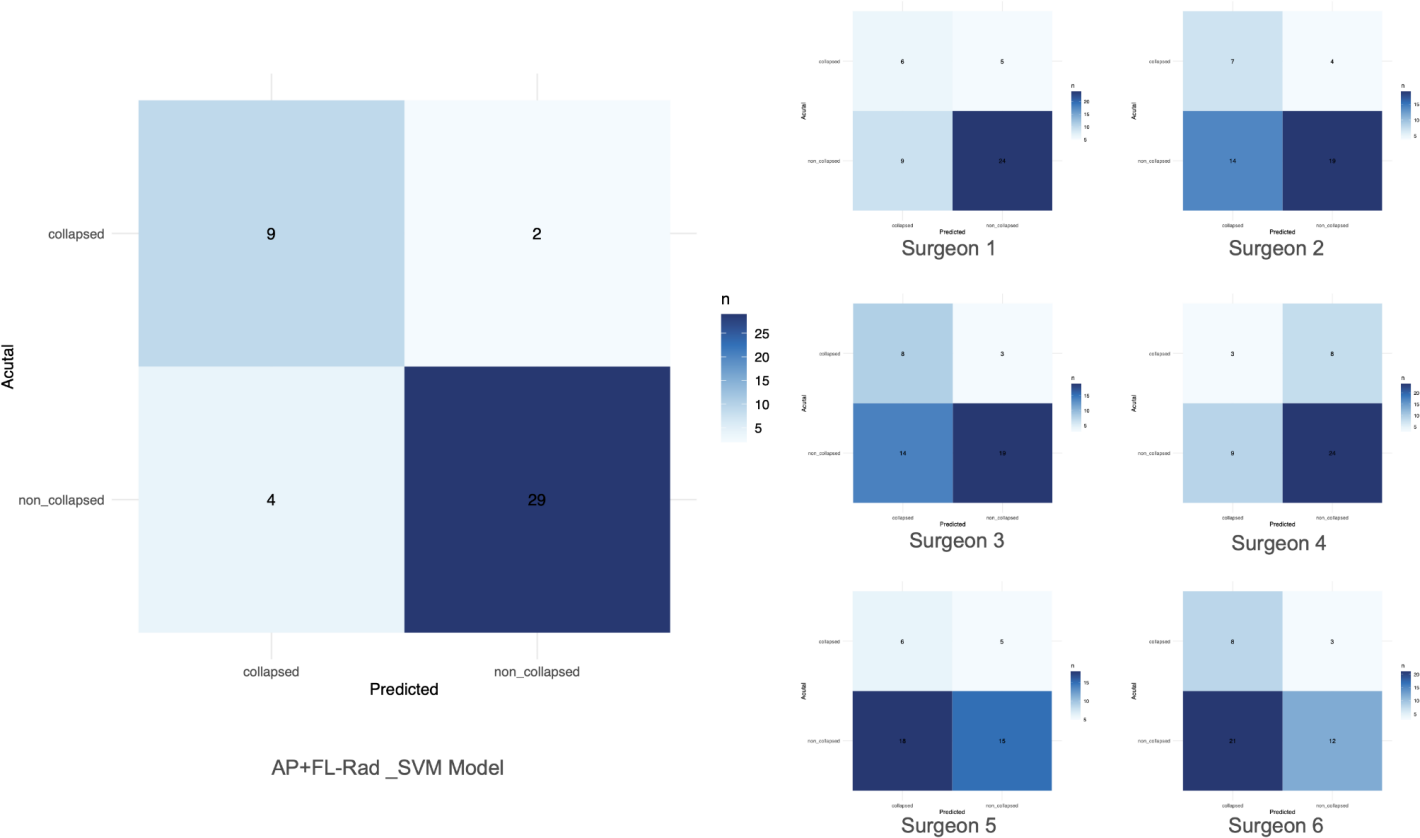
Confusion matrices of the prediction results for the SVM model and each orthopaedic surgeon. The vertical axis represents the actual classification of collapse and non-collapse in the test set, while the horizontal axis represents the predicted classification. The darker the squares, the higher number of cases in which predictions match reality. AP + FL-Rad_SVM: AP + FL combined view model constructed using SVM.

**Fig. 6:**
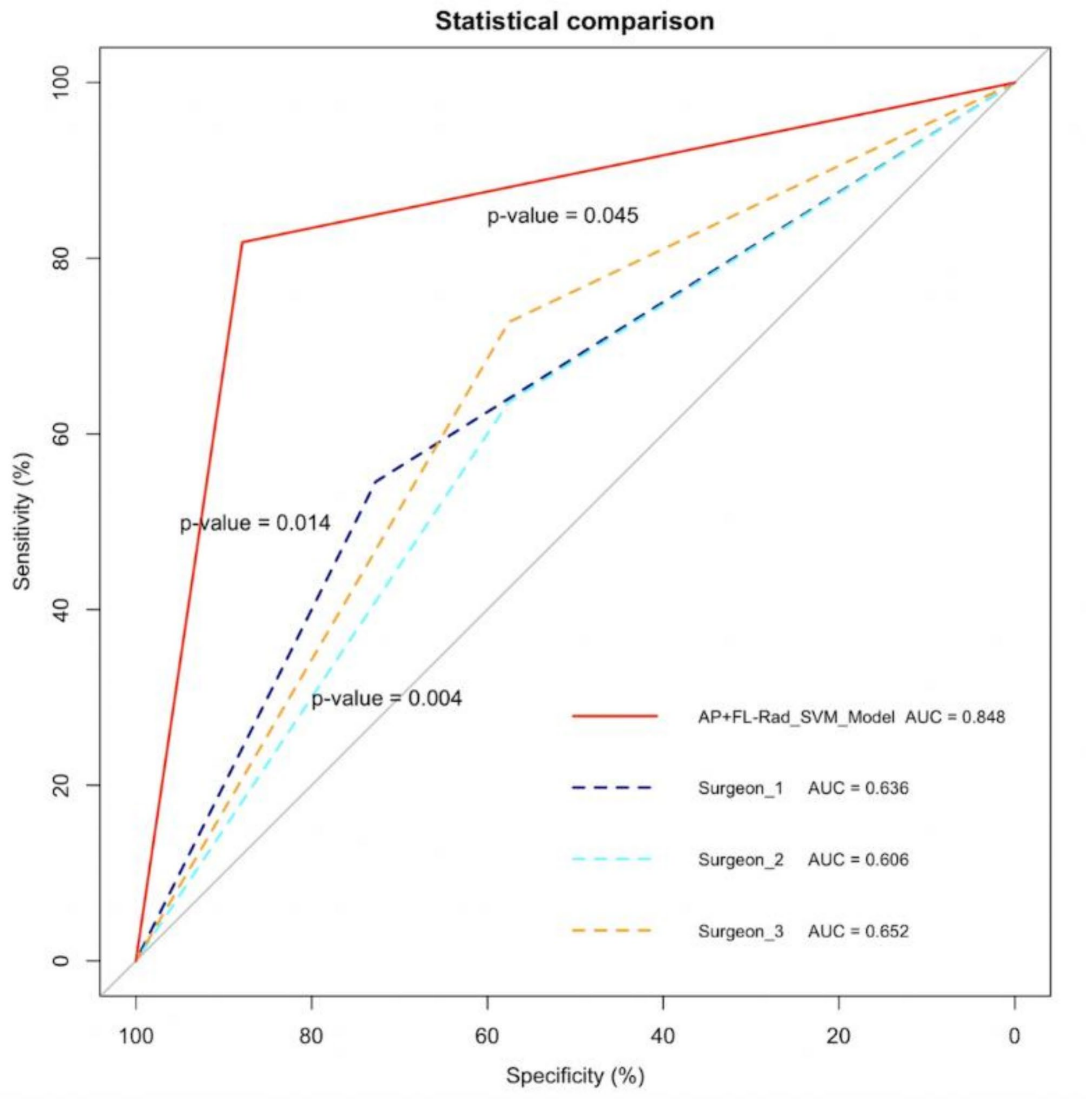
Performance of the AP + FL-Rad_SVM model and attending surgeons on the test set. The y-axis represents sensitivity, and the x-axis represents specificity. The *p*-values in the figure represent comparisons between the AUC of different orthopaedic surgeons and the SVM model. AP + FL-Rad_SVM: AP + FL combined view model constructed using SVM.

An optimal operating threshold value of 0.591 (assessed by maximizing Youden’s index) was used for the ROC curve construction of the optimal model (Supplementary Fig. S2 online). Figure 5 shows the confusion matrices of the prediction results for each orthopaedist and the optimal prediction model. DeLong’s test was used to detect the differences in AUC between the model and three attending surgeons. As shown in Fig. 6, the SVM model significantly outperformed three attending surgeons (*p*-values were 0.014, 0.004, and 0.045 for the comparison of the SVM model to three orthopaedic surgeons, respectively).

## Discussion

In this study, we trained a radiomics SVM model based on AP + FL combined view of hip joint X-ray images to effectively predict femoral head collapse in ARCO stage II ONFH. The model outperformed attending orthopaedic surgeons in predictive ability.

Given that preserving the femoral head is a priority, especially in young patients, accurately predicting femoral head collapse is crucial for selecting appropriate treatments. Previous studies have proposed several risk indicators observable on hip joint images that significantly impact early femoral head collapse, such as ONFH staging and classification^16^, necrotic lesion range > 30%^20,21^, lesion areas in the anterolateral femoral head^18,22,23^, and sclerosis band proportion < 30%^24^. However, current imaging prognostic markers lack clinical feasibility due to multidimensional parameter measurement challenges and highly dependent on clinicians’ experience and judgment, leading to poor prediction consistency. As our study shows, the Cohen’s Kappa values between different doctors remain < 0.5, indicating significant differences in collapse prediction and judgment among clinicians. Unlike the clear imaging presentations of ONFH diagnosis, current prediction methods for femoral head necrosis collapse remain complex and lack stability in clinical application, making them difficult to generalize. Therefore, more accurate, objective and simple methods are urgently needed.

Thanks to the rapid development of artificial intelligence, radiomics and machine learning have shown exceptional capabilities in diagnosing and treating bone and joint diseases in recent years. Using annotated image data, some artificial intelligence algorithms have achieved excellent performance in various hip joint disease fields. The application of artificial intelligence in ONFH diagnosis, staging, and classification has been increased annually and shown excellent performance^12–14^. Currently, most machine learning models for ONFH focus on diagnosis and classification. Chen et al.^25^ proposed a deep learning model using AP and FL X-ray to predict the efficacy of non-vascularized fibular grafting in ONFH, achieving good predictive performance. In predicting the risk of collapse in non-traumatic ONFH with natural progression, Hernigou^26^ developed a machine learning algorithm based on osteonecrosis-related variables from early non-traumatic femoral heads, reported accuracy = 0.712 in 24-month collapse prediction. It was inferior to ours. This result may be due to the inherent selection bias in manually-curated clinical features.

Our study primarily trained X-ray based radiomics machine learning models to predict the collapse of early-stage ONFH. Radiomics, based on original images and utilizing intelligent computation to build models, objectively reflects potentially relevant predictive information according to the internal structure of the femoral head, providing valuable predictive references. Although deep learning has demonstrated remarkable capabilities in medical imaging, traditional machine learning methods still hold significant value. Recent studies^27,28^ have shown that traditional machine learning methods can achieve comparable or even superior performance to deep learning models in certain tasks, particularly for limited datasets or straightforward problems. In terms of imaging modality selection, while MRI offers greater sensitivity in detecting early-stage lesions, X-ray maintains crucial clinical utility due to its accessibility and ability to well reveal noticeable bone changes in the ARCO II stage ONFH. Therefore, the radiomics model developed using X-ray images can offer clinicians a rapid and low-cost diagnostic aid, assisting them in better forecasting the risk of collapse due to ONFH.

Given the critical importance of clinical interpretability of radiomics features in medical applications, SHAP values were adopted into the final model. Our study shows that Shape_Maximum2DDiameterColumn (AP view), Shape_MeshVolume (AP view) and Shape_Maximum2DDiameterRow (FL view) contribute most to the final collapse prediction. These features, derived from shape analysis of the ROI in medical images. Shape_Maximum2DDiameterColumn (AP view) represents the maximum two-dimensional diameter of the necrotic area in the AP view, with larger value indicating more extensive or irregular necrotic lesions. Shape_MeshVolume (AP view) measures the 3D mesh volume enclosing the ROI in the AP view, providing an estimate of the overall size of the necrotic area. Larger mesh volumes typically indicate more extensive lesions. Similar to Shape_Maximum2DDiameterColumn (AP view), Shape_Maximum2DDiameterRow (FL view) measures the maximum 2D diameter of the lesion in the FL view, providing additional information about the lesion’s extent from a different perspective. SHAP plots suggest that larger values of these three features are more prone to femoral head collapse, consistent with previous studies^17,20,26,29,30^. Original_Firstorder_Maximum(AP view) belongs to the first-order statistics category and reflects the brightest pixel value observed within the analyzed image region, typically associated with areas of highest intensity signal in the image. In the context of ONFH, this may correlate with the formation of a necrotic-viable interface (Sclerosis Band) within the femoral head. Referring to SHAP plots, as the value of Original_Firstorder_Maximum (AP view) increases, the probability of femoral head collapse decreases. This suggests that the formation of a sclerosis band may delay femoral head collapse to some extent. In addition, gray-scale texture features, mainly comprising GLDM features, also achieve high scores in predicting collapse risk. Larger feature values indicate more homogeneous texture in regions with high gray values in the image. Based on these findings, structural abnormalities in the necrotic area, such as blood stasis, edema, trabecular fractures, and cystic changes, may also significantly impact collapse occurrence.

Previous studies^17–19,22,25^ have confirmed that both anterior and lateral necrotic lesions of the femoral head are significant prognostic factors for collapse. The combination of X-ray AP and FL views effectively displays the anterior and lateral boundaries of the femoral head, which are highly valuable for predicting femoral head collapse. Meanwhile, our results showed that the femoral head collapse prediction model constructed by combining AP and FL views exhibited better comprehensive prediction results than models using single-view image features alone in both training and test sets. By integrating information from both the AP and FL views, our radiomics signature offers a more holistic assessment of the femoral head’s structural integrity and combines more anatomically complementary features to enhance the generalization ability of the model. This also confirms previous findings.

Considering their widespread use and proven performance in related fields^31–34^, SVM, RF, and SGD were selected for our study. The RF exhibited overfitting tendencies, as it performed best in the training set but exhibited significantly reduced prediction performance in the test set (training vs. test AUC: 0.952 vs. 0.826). Although the calibration curve of the AP + FL-Rad_SVM model does not fit the diagonal perfectly, it still shows a higher match between the model’s predicted probabilities and the actual probabilities. Considering that current calibration issues may be affected by data imbalances from different institutions, the model still demonstrates strong discriminative and generalization capabilities, as shown by the high and close AUC values in both the training and test sets. To ensure the stability and generalization of the radiomics model, we ultimately selected the AP + FL-Rad_SVM model as the optimal radiomics model, which achieved excellent performance in predicting femoral head collapse in ARCO stage II ONFH and demonstrated a significant advantage over inexperienced orthopaedic surgeons.

This study has several limitations. First, plain radiographs cannot identify ARCO stage I necrosis; therefore, our dataset is relatively small, as we only included ONFH with ARCO stage II. No independent validation dataset was established, and the model’s generalization performance has not been fully validated. Second, we only used plain radiographs of the hip joint in this study, while CT and MRI scans may provide more imaging information. Further studies will explore and validate whether CT- and MRI-based imaging information can improve model performance. Finally, manual segmentation of ROI remains inevitably subjective. We hope to achieve complete automation through deep learning in the future. A prospective multi-center study is needed to provide more robust clinical performance.

## Conclusion

In conclusion, our study proposed various machine learning models based on X-ray AP and FL views. The SVM model exhibited the optimal predictive performance and may serve as a potential predictive tool for collapsed risk, assisting in the selection of appropriate hip preservation strategies in the clinical decision-making processes of early ONFH patients. Although our model presented promising results, further studies using large-scale external test data are still required to investigate the model’s efficacy in real-world settings.

## Electronic supplementary material

Below is the link to the electronic supplementary material.

Supplementary Material 1

Supplementary Material 2

Supplementary Material 3

Supplementary Material 4

## Data Availability

The datasets generated or analyzed during the study are available from the corresponding author by reasonable request.
