# Supplementary figures and images for: X-ray based radiomics machine learning models for predicting collapse of early-stage osteonecrosis of femoral head
Source: Sci Rep. 2025 Apr 20;15:13646. doi: 10.1038/s41598-025-94878-2 (PMC12010002; doi:10.1038/s41598-025-94878-2)

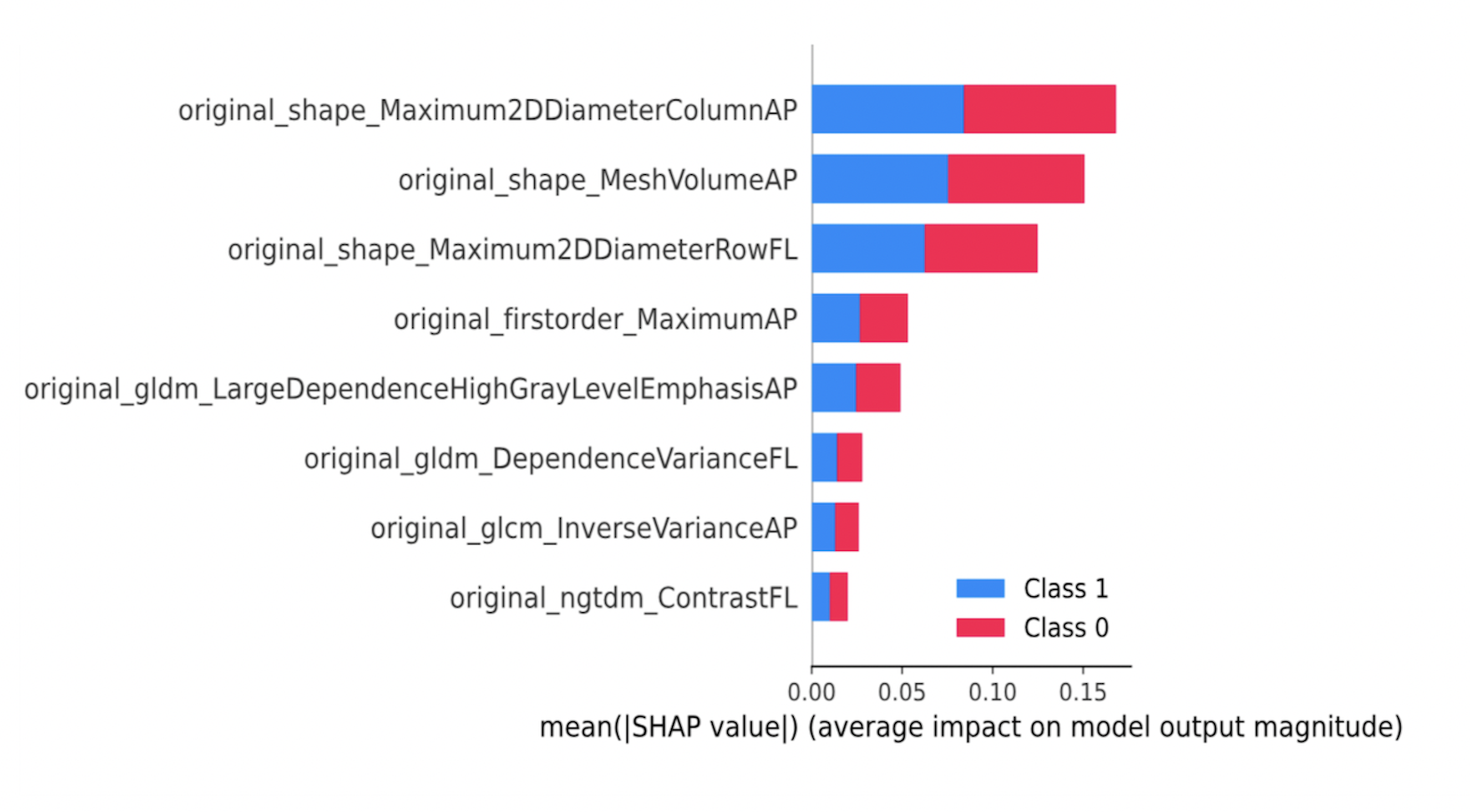

Supplement: Supplementary file 1 — Supplementary Material 1 [file 41598_2025_94878_MOESM1_ESM.tiff]

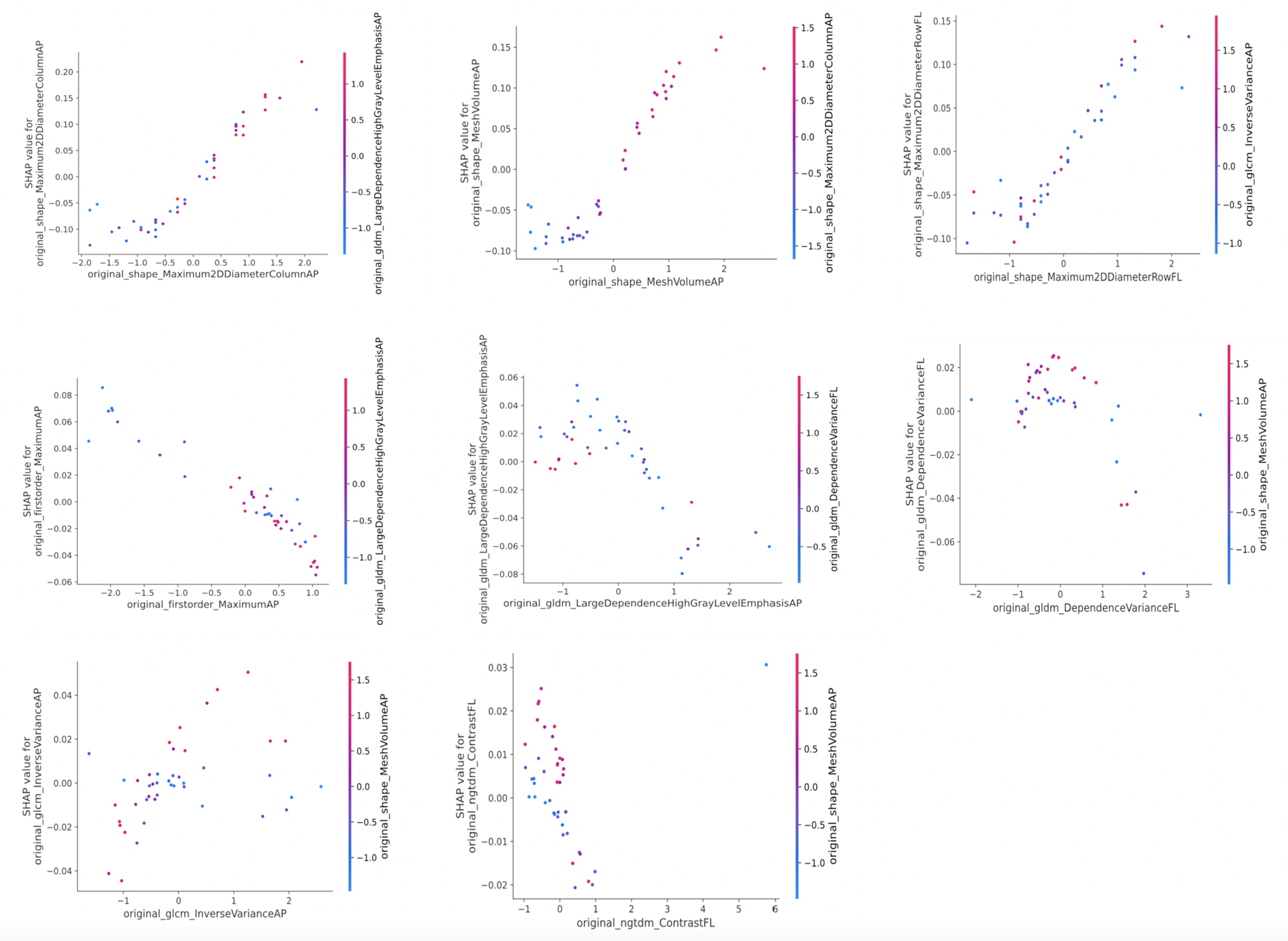

Supplement: Supplementary file 2 — Supplementary Material 2 [file 41598_2025_94878_MOESM2_ESM.tiff]

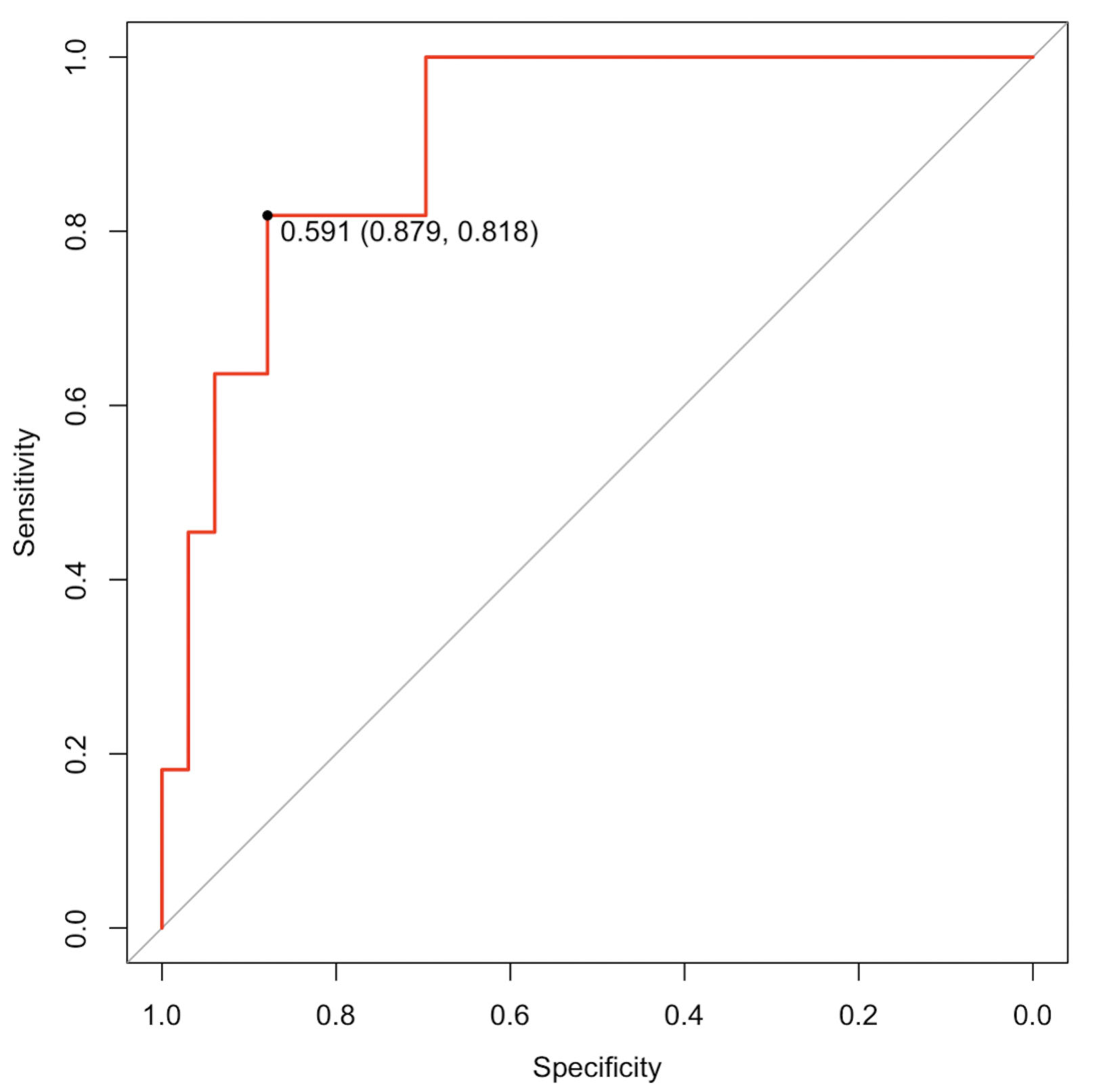

Supplement: Supplementary file 3 — Supplementary Material 3 [file 41598_2025_94878_MOESM3_ESM.tiff]
