# Supplementary material for: X-ray based radiomics machine learning models for predicting collapse of early-stage osteonecrosis of femoral head
Source: Sci Rep. 2025 Apr 20;15:13646. doi: 10.1038/s41598-025-94878-2 (PMC12010002; doi:10.1038/s41598-025-94878-2)
Supplement: Supplementary file 4 — Supplementary Material 4 [file 41598_2025_94878_MOESM4_ESM.pdf]

Supplementary Tab. S1

List of radiomic features extracted by PyRadiomics software package

Shape	First Order	GLCM	GLRLM
Elongation	10Percentile	Autocorrelation	GrayLevelNonUniformity
Flatness	90Percentile	ClusterProminence	GrayLevelNonUniformityNormalized
LeastAxisLength	Energy	ClusterShade	GrayLevelVariance
MajorAxisLength	Entropy	ClusterTendency	HighGrayLevelRunEmphasis
Maximum2DDiameterColumn	InterquartileRange	Contrast	LongRunEmphasis
Maximum2DDiameterRow	Kurtosis	Correlation	LongRunHighGrayLevelEmphasis
Maximum2DDiameterSlice	Maximum	DifferenceAverage	LongRunLowGrayLevelEmphasis
MeshVolume	MeanAbsoluteDeviation	DifferenceEntropy	LowGrayLevelRunEmphasis
MinorAxisLength	Mean	DifferenceVariance	RunEntropy
Sphericity	Median	Id	RunLengthNonUniformity
SurfaceArea	Minimum	Idm	RunLengthNonUniformityNormalized
SurfaceVolumeRatio	Range	Idmn	RunPercentage
VoxelVolume	RobustMeanAbsoluteDeviation	Idn	RunVariance
	RootMeanSquared	Imc1	ShortRunEmphasis
	Skewness	Imc2	ShortRunHighGrayLevelEmphasis
	TotalEnergy	InverseVariance	ShortRunLowGrayLevelEmphasis
	Uniformity	JointAverage	
	Variance	JointEnergy	
		JointEntropy	
		MCC	
		MaximumProbability	
		SumAverage	
		SumEntropy	

		SumSquares	

GLSZM	NGTDM	GLDM
GrayLevelNonUniformity	Busyness	DependenceEntropy
GrayLevelNonUniformityNormalized	Coarseness	DependenceNonUniformity
GrayLevelVariance	Complexity	DependenceNonUniformityNormalized
HighGrayLevelZoneEmphasis	Contrast	DependenceVariance
LargeAreaEmphasis	Strength	GrayLevelNonUniformity
LargeAreaHighGrayLevelEmphasis		GrayLevelVariance
LargeAreaLowGrayLevelEmphasis		HighGrayLevelEmphasis
LowGrayLevelZoneEmphasis		LargeDependenceEmphasis
SizeZoneNonUniformity		LargeDependenceHighGrayLevelEmphasis
SizeZoneNonUniformityNormalized		LargeDependenceLowGrayLevelEmphasis
SmallAreaEmphasis		LowGrayLevelEmphasis
SmallAreaHighGrayLevelEmphasis		SmallDependenceEmphasis
SmallAreaLowGrayLevelEmphasis		SmallDependenceHighGrayLevelEmphasis
ZoneEntropy		SmallDependenceLowGrayLevelEmphasis
ZonePercentage		
ZoneVariance		

GLCM: gray level co-occurrence matrix

GLRLM: gray level run length matrix

GLSZM: gray level size zone matrix

NGTDM: neighboring gray tone difference matrix

GLDM: gray level dependence matrix

Supplementary Tab. S2

Optimized parameters of each model

**Sequence**	**Classifier**	**Optimize parameters**
AP-Rad	SVM	C:0.30559282 gamma:scale kernel:rbf
FL-Rad	SVM	C: 173.957233038677 gamma:scale kernel:rbf
AP+FL-Rad	SVM	C: 0.740447 gamma:scale kernel:rbf
AP-Rad	RF	max_depth:1 min_samples_split:2 n_estimators:71
FL-Rad	RF	max_depth:48 min_samples_split:12 n_estimators:200
AP+FL-Rad	RF	max_depth:200 min_samples_split:18 n_estimators:74
AP-Rad	SGD	Alpha:0.136062441loss: log_loss penalty: elasticnet
FL-Rad	SGD	Alpha:0.022089362loss: log_loss penalty: elasticnet
AP+FL-Rad	SGD	Alpha:0.14044278 loss: log_loss penalty: elasticnet

AP-Rad: AP view model radiomics feature datasets

FL-Rad: FL view model radiomics feature datasets

AP+FL-Rad: AP+FL combined view model radiomics feature datasets

SVM: support vector machine

RF: random forest

SGD: stochastic gradient descent

Supplementary Fig. S1

SHAP method interpretation of the global model

(a) SHAP summary bar chart.

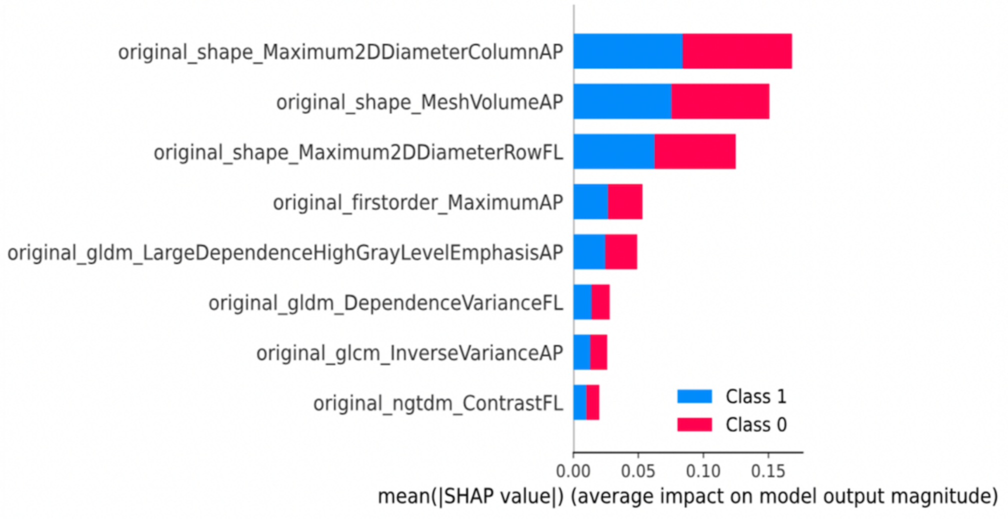

The vertical axis represents radiomics features, and the horizontal axis stands for SHAP value. The features are ranked from top to bottom according to the average impact of different features on the output magnitude of the model. The figure shows the importance of the features from top to bottom. The top 3 important features were original_shape_Maximum2DDiameterColumnAP, original_shape_MeshVolumeAP, and original_shape_Maximum2DDiameterRowFL.

Class 0: non-collapsed

Class 1: collapsed

(b) Partial Dependence Diagram (PDP).

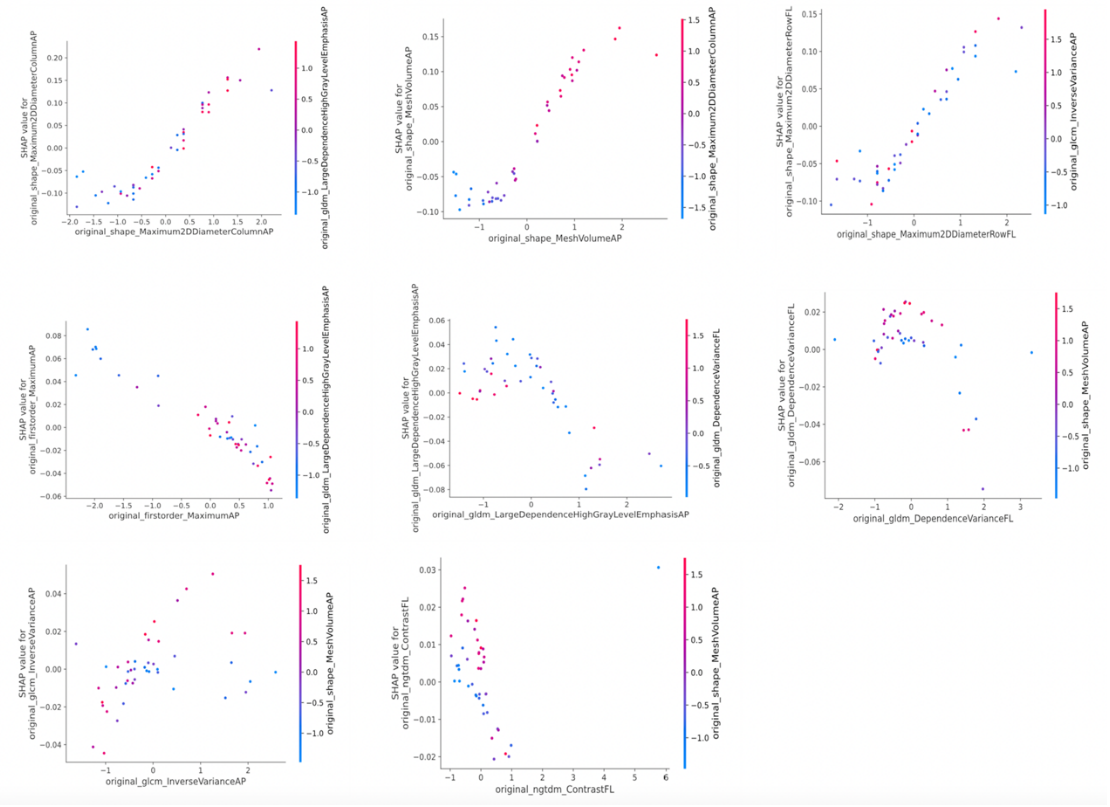

The vertical axis represents the SHAP value, the horizontal axis represents different radiomics features. Each point represents a radiomics feature. The PDP plot showed the marginal effect of one or two features on the prediction results of machine learning, and also reflected the relationship between SHAP values and features, such as linear, monotonous or other more complex relationships. For example, there was a linear correlation between original_shape_MeshVolumeAP and SHAP values. As shown in the PDP plot, the greater the feature value of original_shape_MeshVolumeAP, the higher the SHAP value, and the more likely the femoral head was to collapse.

Supplementary Fig. S2

ROC curve analysis of AP+FL-Rad_SVM model on test set

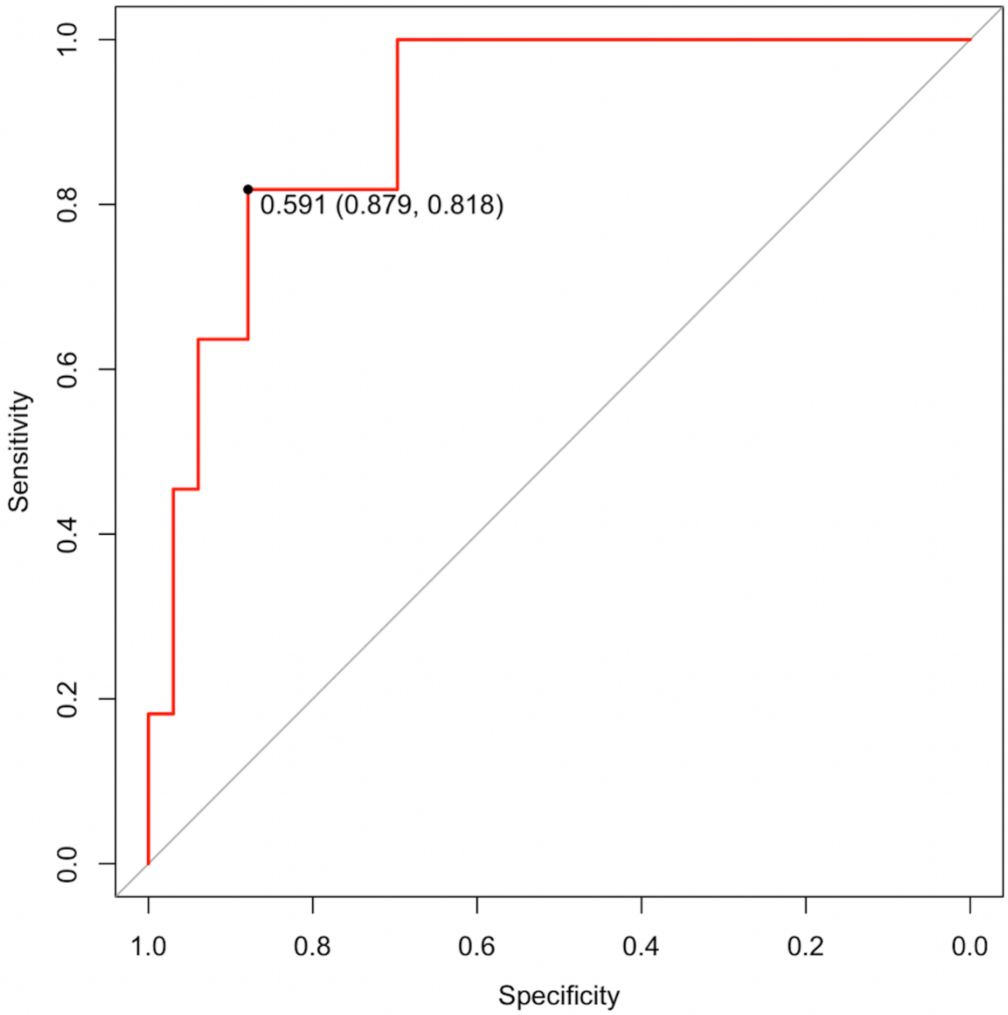

The ordinate is the sensitivity, and the abscissa is the specificity. The point in this figure represents the best prediction threshold of 0.591 when the Youden's index is the largest (the corresponding prediction model specificity and sensitivity are 0.879 and 0.818, respectively).
